# Artificial light at night alters the relative dominance of invasive plants in a nutrient-dependent manner across native species richness levels

**DOI:** 10.1098/rspb.2026.0507

**Published:** 2026-10-28

**Authors:** Haobin Zhang, Ayub M. O. Oduor, Lu Xiao, Yanjie Liu

**Affiliations:** ^1^ Jilin Da'an Agro-ecosystem National Observation and Research Station, Key Laboratory of Marsh Wetland Ecosystem Conservation and Restoration, National Forestry and Grassland Administration, State Key Laboratory of Black Soils Conservation and Utilization, Northeast Institute of Geography and Agroecology, Chinese Academy of Sciences, Changchun, Jilin, People's Republic of China; ^2^ Department of Biological and Life Sciences, Technical University of Kenya, Nairobi, Kenya; ^3^ Shandong Key Laboratory of Eco-Environmental Science for Yellow River Delta, Shandong University of Aeronautics, Binzhou, Shandong, People's Republic of China

**Keywords:** artificial light at night, native-community species richness, non-native plant invasion, nutrient enrichment, fluctuating resource hypothesis

## Abstract

Non-native plant invasions pose a global challenge, yet how nutrient enrichment and artificial light at night (ALAN) interact with native-community species richness to shape invasive-plant performance and community-level dominance remains unclear. We conducted a multispecies greenhouse experiment in which target invasive species and native communities were established simultaneously under fully crossed factorial combinations of ALAN (No-ALAN or ALAN), nutrient enrichment (low or high) and native species richness (two, four or eight species). We quantified target invasive-plant above-ground biomass, flowering probability and proportional above-ground biomass, as well as native-community and total-community above-ground biomass. Nutrient enrichment increased invader above-ground biomass by 272.88%, flowering probability by 30.39% and native-community above-ground biomass by 97.58%, although its effect on proportional invader biomass remained uncertain. ALAN did not clearly affect invader biomass or flowering but increased its proportional biomass under high-nutrient conditions by 5.74% and reduced it under low nutrients by 4.75%. Native species richness showed no clear effects on invader performance, community biomass or responses to ALAN and nutrients. Within this experimental context, invasive-plant performance was driven primarily by nutrient enrichment, with ALAN facilitating invader dominance under high-nutrient conditions, indicating that invasion outcomes were more influenced by resource availability than by native-community species richness.

## 1. Introduction

Biological invasion by non-native plant species has emerged as a major global challenge, posing significant threats to native biodiversity, human health and economic systems [1–3]. Effective mitigation of these threats hinges on a deep understanding of the mechanisms underlying non-native plant invasions [4]. Among the environmental factors that can drive non-native plant invasions, nutrient enrichment and artificial light at night (ALAN) are increasingly widespread components of global environmental change. Nutrient enrichment can relax below-ground resource limitation, whereas ALAN modifies the above-ground light environment; both can influence invasive plant growth and competitive interactions [5–8]. The fluctuating resource hypothesis posits that increases in resource availability create opportunities for invasive plants to establish and spread [9–11]. These opportunities may be amplified when invasive plants capture and utilize newly available resources more effectively than native species, thereby gaining a competitive advantage [12–14]. Extensive empirical evidence supports this hypothesis, particularly regarding nutrient enrichment. Greenhouse experiments consistently demonstrate that elevated nutrient availability disproportionately increases biomass of invasive plants relative to native species [11,15–17]. This pattern is further supported by large-scale field studies showing that invasive plants benefit more from nutrient addition than their native counterparts [5]. While below-ground resource changes, particularly nutrient enrichment, have been widely studied as drivers of plant invasion, the role of above-ground resource alterations remains poorly understood. Thus, although nutrient enrichment is an established driver of plant invasion and evidence for ALAN as an invasion driver is emerging, their interactive effects on invasive-plant performance and community-level dominance remain poorly understood [7,8,18].

Artificial light at night is now widely recognized as an important ecological driver of global environmental change [19–21]. ALAN encompasses a broad continuum of exposure, ranging from diffuse urban skyglow, typically approximately 0.01–0.1 lux under clear skies, to substantially brighter direct illumination near artificial light sources [22]. At the brighter end of this continuum, ALAN may function not only as an altered environmental condition but also as an additional light resource capable of influencing plant carbon gain and growth [23,24]. Although the effects of ALAN on plant phenology, growth, reproduction and resource allocation are well documented [24,25], research explicitly addressing its role in plant invasions has only recently begun, following the pioneering work of Speißer *et al.* [6]. Invasive plants are often characterized by high phenotypic plasticity and efficient resource acquisition [26,27], traits that may confer a competitive advantage under altered night-time light conditions. Consistent with this expectation, emerging evidence indicates that ALAN promotes the growth and performance of invasive plants more strongly than that of native species, thereby increasing invasion risk [6,28–31]. While ALAN and nutrient enrichment have each been shown to independently facilitate plant invasions, their interactive effects remain poorly understood. Addressing this gap is particularly important because invasive plants commonly establish in disturbed urban environments, where artificial night-time lighting and elevated nutrient availability frequently co-occur [32].

Light availability, encompassing both daytime irradiance and ALAN, can interact with nutrient enrichment to influence plant invasion because increases in these resources generally stimulate physiological activity and growth [33,34]. Invasive plants often show stronger growth responses to resource enrichment than native species [14,35]. For example, cross-regional experiments in China and Germany showed that simultaneous increases in daytime light and nutrient availability substantially enhanced the competitive dominance of invasive plants [36]. More recent evidence further suggests that ALAN can act synergistically with nutrient enrichment, giving invasive species disproportionate growth advantages over co-occurring native species and thereby increasing invasion risk [8]. However, these insights are largely derived from single-species or pairwise studies, which do not fully capture the multispecies interactions that characterize natural plant communities. This limitation constrains predictions of how ALAN and nutrient enrichment interact to shape invasion dynamics under more realistic multispecies community contexts.

Beyond their direct effects on plant growth, ALAN and nutrient enrichment may also influence invasion dynamics indirectly by altering the strength of biotic interactions within resident communities. Biotic interactions play a central role in shaping plant invasion processes under global change [7,37]. According to the diversity–invasibility hypothesis, species-rich communities should be more resistant to invasion by non-native plants [38–40]. This resistance arises because diverse native assemblages more completely partition and use available resources across spatial and temporal gradients, thereby increasing competitive pressure on invasive species during establishment [41,42]. Species-rich communities are also more likely to contain functionally similar native species that compete directly with invaders for limiting resources through sampling effects [43,44]. Consistent with this expectation, empirical studies have shown that invasive plants establish more readily and achieve greater dominance in low-diversity communities, where weaker biotic resistance and greater resource availability create opportunities for invasion [41,45,46]. However, this biotic resistance can be significantly altered by nutrient enrichment, one of the most pervasive drivers of global environmental change [47]. By relaxing resource limitation, nutrient enrichment can disproportionately enhance the growth and competitive ability of fast-growing invasive plants and reduce native species diversity through competitive exclusion [5,48]. Similarly, ALAN may extend photosynthetic activity and alter competitive interactions in ways that favour resource-responsive invaders. Consequently, ALAN and nutrient enrichment may weaken, or even reverse, the negative diversity–invasibility relationship by relaxing resource limitation and amplifying competitive asymmetries between invasive plants and native resident communities. Yet, whether ALAN and nutrient enrichment interact to weaken biotic resistance and reshape the diversity–invasibility relationship in multispecies plant communities remains unknown.

Given the limited understanding of invasion dynamics under co-occurring ALAN and nutrient enrichment in multispecies communities, we conducted a greenhouse experiment to test how these drivers interact to influence target invasive-plant performance and community-level dominance, and whether native-community species richness moderates these responses through biotic resistance. Our study focused on relatively high-intensity, direct ALAN rather than diffuse skyglow. Specifically, we tested whether ALAN, nutrient enrichment and native-community richness jointly shaped target invasive-plant performance. We predicted that: (i) ALAN and nutrient enrichment would interact synergistically to increase target invasive-plant absolute above-ground biomass, flowering probability and proportional above-ground biomass beyond their independent effects; (ii) greater native species richness would suppress these responses through stronger biotic resistance; and (iii) combined ALAN and nutrient enrichment would weaken richness-mediated suppression by alleviating light and nutrient limitation and thereby reducing competition-based resistance from native communities.

## 2. Material and methods

### (a) Study species

To test the individual and interactive effects of ALAN and nutrient enrichment on the performance and proportional dominance of invasive plant species across different native species richness levels, we selected 8 invasive non-native plant species as targets and 10 native plant species to assemble 15 native communities (supplementary material, table S1). The eight invasive species represented seven genera across five families, thereby minimizing phylogenetic bias [49] and ensuring broader taxonomic representation. The 10 native species used to assemble the native communities represented nine genera across eight families (supplementary material, table S1). These species commonly coexist in disturbed habitats, including urban and peri-urban areas exposed to ALAN (L.X. 2023, personal observation). Species were classified as native or invasive using China as the focal geographic region, with native status determined based on the online Flora of China database (www.efloras.org). Because the term ‘invasive’ is used differently across studies, we follow Colautti & MacIsaac [50] in defining ‘invasive species’ here as non-native plant species that have established, spread beyond their introduction sites, cause negative ecological impacts and are listed as invasive alien plants in China [51]. Because the experiment was conducted in pots under greenhouse conditions, the assembled native communities should not be interpreted as intact natural plant communities. Instead, they represent experimentally assembled mixtures of species native to China, allowing us to test whether increasing native species richness modifies the performance and dominance of target invasive plants under ALAN and nutrient enrichment. All seeds were collected from wild populations, except those of *Lolium perenne*, which were purchased from a commercial seed supplier (Green Alliance Ecological Enterprise Shop, Changde, Hunan, China).

### (b) Experimental artificial light at night facility

To implement the ALAN treatment, we established eight greenhouse compartments, each measuring 2.4 m^2^, with distances of 0.3–1.2 m between compartments. The compartments were arranged on three benches in a greenhouse at the Northeast Institute of Geography and Agroecology, Chinese Academy of Sciences (125°24′03″ E, 43°59′49″ N; supplementary material, figure S1). Four compartments were randomly assigned to the ALAN treatment, while the remaining four served as No-ALAN controls. For the ALAN treatment, compartments were illuminated using LED spotlights (5 W, DC 12–24 V, cool white, 6500 K), regulated by photoelectric switches (XT KG-F, Zhejiang Xintuo New Energy Co., Yueqing, Zhejiang, China). The lights automatically turned on when ambient greenhouse light intensity, measured by a photoelectric sensor positioned outside the illuminated compartments and shielded from light emitted by the ALAN lamps, fell below 30 lux at sunset. They turned off when ambient light intensity rose above 30 lux at sunrise. To ensure even ALAN distribution and stable lighting conditions for the plants, each compartment was divided into two sub-compartments measuring 1 × 1.2 m. One spotlight was positioned 1 m above the centre of each sub-compartment and attached to a metal frame with a lampshade that directed light downward. To simulate ground-level street lighting, light intensity was adjusted using a dimmer (DC 12–24 V, 30 A, Shenzhen Gepute Technology Co., China) to an average of 30.48 ± 0.21 lux (mean ± s.d.) within the compartments. This intensity falls at the relatively high-intensity, direct-illumination end of the ALAN continuum and closely matches the 30-lux treatment used to represent high-intensity street lighting in recent plant experiments [23]. Its illuminance is approximately 300–3000 times that of typical clear-sky urban skyglow (0.01–0.1 lux); therefore, our treatment should be interpreted as direct, streetlight-like exposure rather than diffuse landscape-level skyglow. To minimize potential artefacts caused by the lighting apparatus, metal frames and lampshades without LED lights were also installed in the No-ALAN compartments. From sunset to sunrise, the sides and top of the greenhouse were covered with blackout fabric to prevent external light sources from affecting the experimental lighting conditions.

### (c) Experimental set-up

The experiment employed a fully crossed factorial design with three factors: native species richness (two, four or eight species), ALAN (No-ALAN or ALAN) and nutrient enrichment (low or high). ALAN was applied at the compartment level, with four compartments per light treatment, whereas nutrient enrichment and native species richness were manipulated at the pot level. Each of the 8 invasive species was grown under all 12 treatment combinations, with 5 replicates per species per treatment combination. This resulted in 480 experimental pots in total: 8 invasive species × 3 native species richness levels × 2 ALAN treatments × 2 nutrient enrichment × 5 replicates (supplementary material, figure S1a).

From 20 to 30 August 2023, we sowed seeds of each species separately in plastic trays (diameter: 25.5 cm; height: 4 cm) filled with sterilized peat moss (Pindstrup Plus, Pindstrup Mosebrug A/S, Denmark). To ensure uniform initial seedling sizes at the beginning of the experiment, sowing dates were staggered based on information from previous experiments (supplementary material, table S1). Before sowing, we disinfected the seeds by immersing them in a 0.5% sodium hypochlorite solution for 5 minutes, and subsequently rinsed them thoroughly with deionized water. After sowing, all trays were placed in the greenhouse under natural light, with temperatures ranging from 20°C to 28°C.

On 12 September 2023, we transplanted similarly sized seedlings of the eight target invasive species and 10 native species into 3 l square plastic pots (bottom dimensions: 15.5 × 15.5 cm; top dimensions: 18.0 × 18.0 cm; height: 12.5 cm) filled with a 1 : 1 (v/v) mixture of sand and vermiculite. One seedling of a target invasive species was planted at the centre of each pot, resulting in 480 experimental pots. Eight native individuals were planted around the target individual to establish native communities at three species-richness levels: two, four or eight species. In the 2-species treatment, 2 native species were randomly selected from the 10-species pool, and 4 individuals of each species were planted in each pot. For the four-species treatment, four native species were selected, and two individuals of each species were planted per pot. For the eight-species treatment, eight native species were selected, and one individual of each species was planted per pot. Thus, native planting density was held constant at eight individuals per pot across all richness treatments. Five distinct native-community compositions were used within each richness level (i.e., five composition-level replicates per richness level), and each composition was crossed with all combinations of the ALAN and nutrient treatments and with each target invasive species (supplementary material, figure S1b). The native diversity treatment was designed to manipulate native species richness while holding total native abundance constant under standardized greenhouse conditions. We used this design to test whether experimentally increased native richness altered growth performance of the target invasive species; in interpreting these effects, we did not assume that greater species richness necessarily represented stronger competition, more complete niche occupation or more exhaustive resource use. The 480 pots were distributed across 8 greenhouse compartments, with 4 compartments assigned to ALAN and the remaining 4 to No-ALAN conditions. Each compartment contained 60 pots representing 2 target invasive species, with 30 pots per species. For each target species, these 30 pots represented all combinations of 3 native richness levels and 2 nutrient levels, with 5 replicates per combination. All compartments were uniformly exposed to natural daylight. We replaced all dead seedlings within one week of transplanting and measured the initial height of the target invasive species.

On 21 September 2023, we initiated the ALAN and nutrient enrichment treatments. To establish low- and high-nutrient conditions, each pot received 50 ml of a 20 : 20 : 20 NPK liquid fertilizer solution (Peters Professional, EC Fertilizer) prepared at concentrations of 1 or 4 g l^−1^, respectively, once weekly for nine consecutive weeks. These concentrations were selected based on a previous greenhouse experiment in which they produced contrasting nutrient conditions [36]. Based on the substrate surface area of each pot, calculated from its 18.0 × 18.0 cm square top (approx. 324 cm^2^), the treatments supplied cumulative nitrogen inputs of approximately 2.8 and 11.1 g N m^−2^, equivalent to 28 and 111 kg N ha^−1^, respectively, over the nine-week experiment. These inputs fall within the broad range of nitrogen enrichment measured or experimentally applied in field ecosystems. For example, atmospheric nitrogen deposition approaching 30 kg N ha^−1^ yr^−1^ has been recorded in highly polluted dryland ecosystems, while field grassland experiments have applied approximately 100–105 kg N ha^−1^ yr^−1^ and, in some cases, rates ranging up to 280 kg N ha^−1^ yr^−1^ [52–54]. Nevertheless, because our nutrient inputs were concentrated within nine weeks rather than distributed over an entire year, they should not be interpreted as direct equivalents of annual atmospheric deposition. Instead, the low- and high-nutrient enrichments represent moderate and strong growing-season nutrient enrichment, respectively, relevant to nutrient-polluted habitats. Plants were watered daily, and each pot was placed on an individual plastic dish. Any drainage collected in the dish was used to rewater the same pot. To minimize spatial variation in light and other greenhouse conditions, pots were randomly repositioned within each compartment every week and rotated among compartments assigned to the same light treatment (No-ALAN or ALAN) every three weeks throughout the experiment.

### (d) Measurements

On 17 November 2023 (66 days after transplanting), we recorded the flowering status of the target invasive species in each pot. An individual was classified as flowering when it had at least one visible reproductive structure, including an unopened flower bud, an open flower with an intact corolla or a mature fruit. This classification follows standard phenological approaches in which individuals bearing buds, flowers or fruits are considered to have entered the reproductive phenophase [55]. On 18 November 2023, we separately harvested the above-ground biomass of the target invasive plant and the associated native community in each pot. Six target invasive individuals died during the experiment and were excluded from the statistical analyses; the remaining 474 individuals were harvested and analysed. All harvested plant materials were dried at 65°C for 72 hours and weighed to determine dry biomass. To quantify the relative dominance of the target invasive species, we calculated its proportional above-ground biomass by dividing its above-ground biomass by the total above-ground biomass in the pot: proportional invasive biomass = invasive biomass / (invasive biomass + native-community biomass).

### (e) Statistical analyses

All statistical analyses were conducted in R version 4.5.2 [56]. We used Bayesian hierarchical models fitted with the *brm* function in the *brms* package [57] to test the main and interactive effects of ALAN, nutrient enrichment and native species richness on the performance of the target invasive species and the native community. Fixed effects in the initial full model included ALAN treatment (No-ALAN vs. ALAN), nutrient enrichment (low vs. high), native species richness (two, four or eight species, treated as a continuous predictor) and all possible two-way and three-way interactions among these predictors. We included standardized initial height of the target invasive species as a covariate in all models to account for variation in the initial size of the target individual. Target species was included as a species-level random effect. Native-community identity and greenhouse compartment identity at harvest were also included as random effects to account for variation among native communities and non-independence of plants within the same greenhouse compartment.

Above-ground biomass of the target invasive species and the native community was analysed using lognormal models. The proportional above-ground biomass of the target invasive species was analysed using a beta regression model with a logit link [58]. Flowering probability of the target invasive species was analysed as a binary response using a Bernoulli model with a logit link. As a supplementary analysis, we also analysed total above-ground biomass per pot, calculated as the sum of target invasive and native-community above-ground biomass, using a lognormal model and the same Bayesian model-selection framework.

We determined the final model specification in two steps. First, we compared candidate species-level random-effect structures while retaining the full fixed-effect structure. These candidate models allowed treatment effects to vary among target species by including species-level random slopes for ALAN, nutrient enrichment or their interaction where appropriate. Second, after determining the species-level random-effect structure for each response variable, we compared candidate fixed-effect structures using the same random-effect structure. The full three-way interaction model, corresponding to the complete factorial design, was used as the starting model. We then evaluated whether this full model improved out-of-sample predictive performance relative to simpler models. Candidate fixed-effect structures included the full three-way interaction model, a model containing all two-way interactions but excluding the three-way interaction, a model retaining native species richness as a main effect and the interaction between ALAN and nutrient enrichment and a main-effect only model. Candidate models were compared using approximate leave-one-out cross-validation [59]. When models showed similar expected log predictive density, we selected the simpler model for parsimony [60].

Final model selection was based on model convergence, Pareto-*k* diagnostics, approximate leave-one-out cross-validation and model parsimony. Details of the candidate models, model diagnostics and model comparison results are provided in supplementary material, table S2. Final models were run with four chains of 6000–8000 iterations each, including 2000–3000 warm-up iterations; more iterations were used for complex models to improve sampling stability. Across the final selected models, maximum Rhat values ranged from 1.001 to 1.002, minimum bulk effective sample sizes ranged from 2313 to 6003 and minimum tail effective sample sizes ranged from 1502 to 9714. No divergent transitions occurred after warm-up. Maximum Pareto-*k* values ranged from 0.489 to 0.588, with all values below 0.7. Posterior predictive checks showed good agreement between the observed data distributions and the corresponding posterior predictive distributions [61]. For the final selected models, we report posterior means, posterior standard deviations and 95% credible intervals. Effects were interpreted based on their direction, magnitude and uncertainty; effects whose 95% credible intervals included zero were considered to have substantial posterior uncertainty.

## 3. Results

Nutrient enrichment increased the above-ground biomass of target invasive species by 272.88% (figure 1a; table 1). By contrast, we found no clear evidence that ALAN or native species richness affected above-ground biomass of the target invasive species (table 1). ALAN interacted with nutrient enrichment to alter the proportional above-ground biomass of target invasive species (figure 1b; table 1). ALAN increased proportional above-ground biomass by 5.74% under high-nutrient conditions but decreased it by 4.75% under low-nutrient conditions (figure 1b). Nutrient enrichment had a positive estimated effect on proportional above-ground biomass of target invasive species, although the associated uncertainty was substantial because its 95% credible interval slightly overlapped zero (table 1). The estimated main effects of ALAN and native species richness on proportional above-ground biomass of target invasive species were also uncertain (table 1). Species-specific posterior estimates showed a consistent positive effect of nutrient enrichment on above-ground biomass across all eight target invasive species, whereas the effects of ALAN on their proportional above-ground biomass varied in direction and magnitude (supplementary material, figure S2).

**Figure 1.**
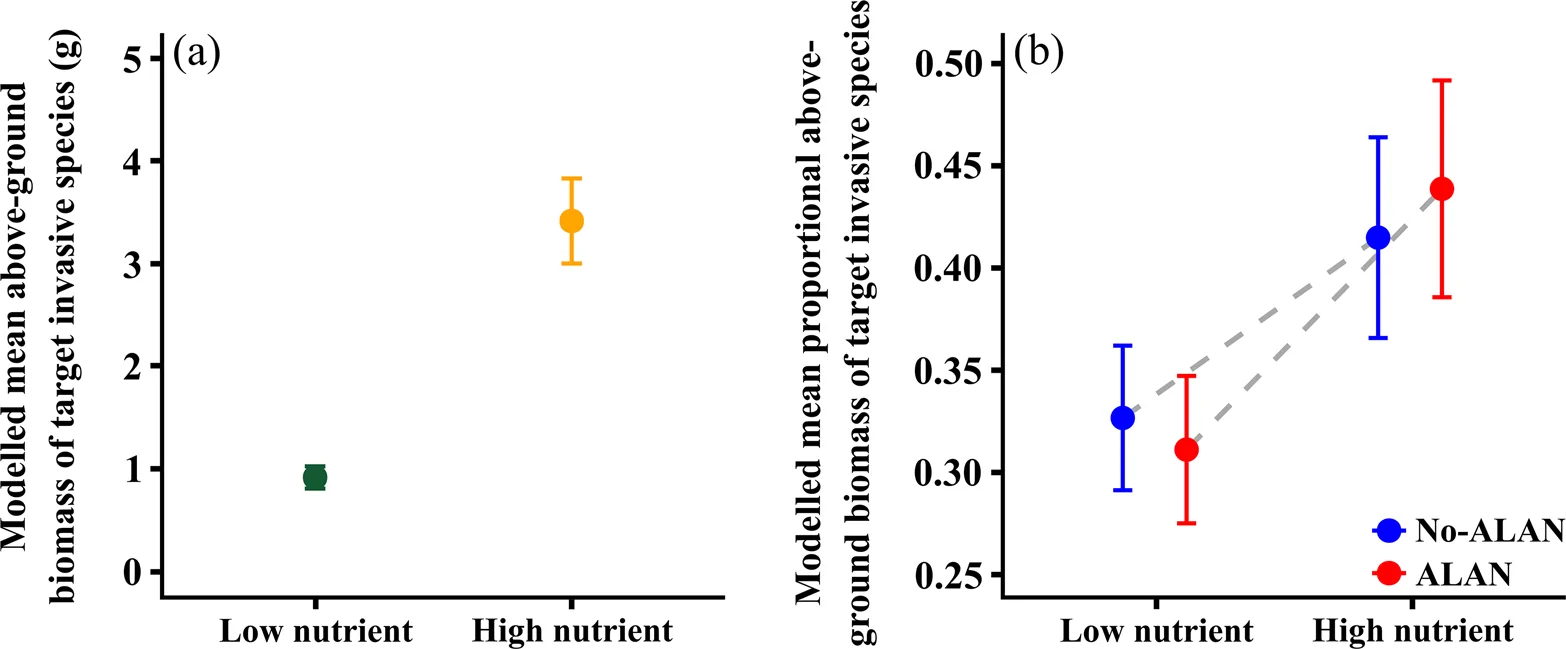
Modelled (a) mean above-ground biomass of target invasive species in response to nutrient enrichment and (b) mean proportional above-ground biomass across these species in response to the interaction between ALAN and nutrient enrichment. Points represent posterior means, and error bars represent 95% credible intervals.

**Table 1.** Results of the final Bayesian hierarchical models testing the effects of artificial light at night (ALAN; L: No-ALAN vs. ALAN), nutrient enrichment (N: low vs. high) and native-community species richness (C: two, four or eight species) on the above-ground biomass and proportional above-ground biomass of target invasive species, the above-ground biomass of native communities and the flowering probability of target invasive species. Posterior means, posterior standard deviations (s.d.), and the lower (LCrI) and upper (UCrI) bounds of the 95% credible intervals (CrIs) are presented. Boldface estimates indicate statistically significant effects, defined here as those for which the 95% CrIs exclude zero.

response variable	term	estimate	posterior s.d.	L95%CrI	U95%CrI
above-ground biomass of target invasive species	intercept (grand mean)	0.198	0.427	−0.654	1.048
	initial height of target invasive species	**0.275**	0.039	0.198	0.353
	ALAN (L)	0.083	0.056	−0.030	0.195
	nutrient enrichment(N)	**0.569**	0.106	0.354	0.777
	species richness of the native community (C)	−0.028	0.032	−0.090	0.037
proportional above-ground biomass of target invasive species	intercept (grand mean)	−0.481	0.531	−1.529	0.586
	initial height of target invasive species	**0.319**	0.047	0.228	0.411
	L	−0.017	0.069	−0.157	0.119
	N	0.236	0.128	−0.021	0.491
	C	−0.034	0.053	−0.138	0.069
	L : N	**0.063**	0.023	0.018	0.107
above-ground biomass of native communities	intercept (grand mean)	0.703	0.197	0.314	1.099
	initial height of target invasive species	**−0.076**	0.022	−0.119	−0.033
	L	**0.093**	0.044	0.004	0.180
	N	**0.324**	0.052	0.219	0.427
	C	0.015	0.027	−0.040	0.068
	L : N	**−0.041**	0.011	−0.062	−0.020
flowering probability of target invasive species	intercept (grand mean)	−0.385	1.670	−3.682	3.048
	initial height of target invasive species	0.497	0.374	−0.221	1.237
	L	−1.869	1.015	−3.876	0.213
	N	**1.354**	0.562	0.319	2.585
	C	−0.009	0.087	−0.183	0.161
	L : N	0.796	0.571	−0.277	2.020

ALAN and nutrient enrichment increased above-ground biomass of the native communities by 18.42% and 97.58%, respectively (figure 2a,b; table 1). The positive effect of ALAN on the above-ground biomass of native communities was greater under low-nutrient (29.75%) than under high-nutrient (13.10%) conditions, indicating that the effect of ALAN depended on nutrient availability (figure 2c; table 1). There was no clear evidence that native species richness affected native-community above-ground biomass (table 1) or total above-ground biomass per pot (supplementary material, table S3). Nutrient enrichment increased the flowering probability of target invasive species by 30.39% (figure 3; table 1). However, we found no clear evidence that ALAN, native species richness or the interaction between ALAN and nutrient enrichment affected flowering probability (table 1). Initial height of the target invasive plants was significantly associated with their above-ground and proportional above-ground biomass and with the above-ground biomass of native communities, whereas its association with flowering probability had substantial posterior uncertainty (table 1).

**Figure 2.**
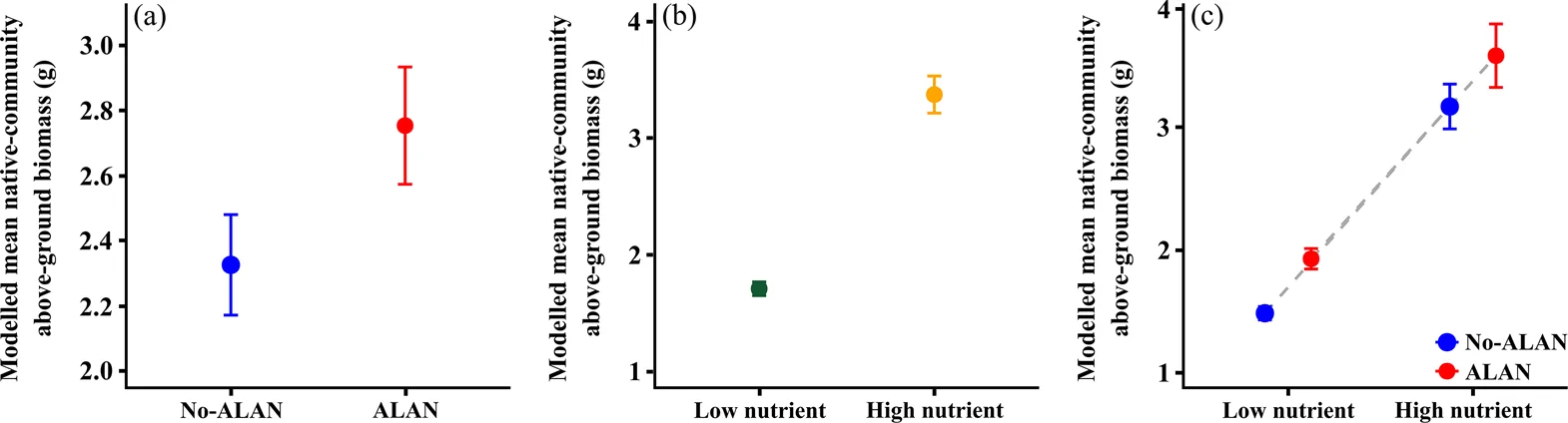
Modelled mean native-community above-ground biomass in response to (a) ALAN, (b) nutrient availability and (c) their interaction. Points represent posterior means, and error bars represent 95% credible intervals.

**Figure 3.**
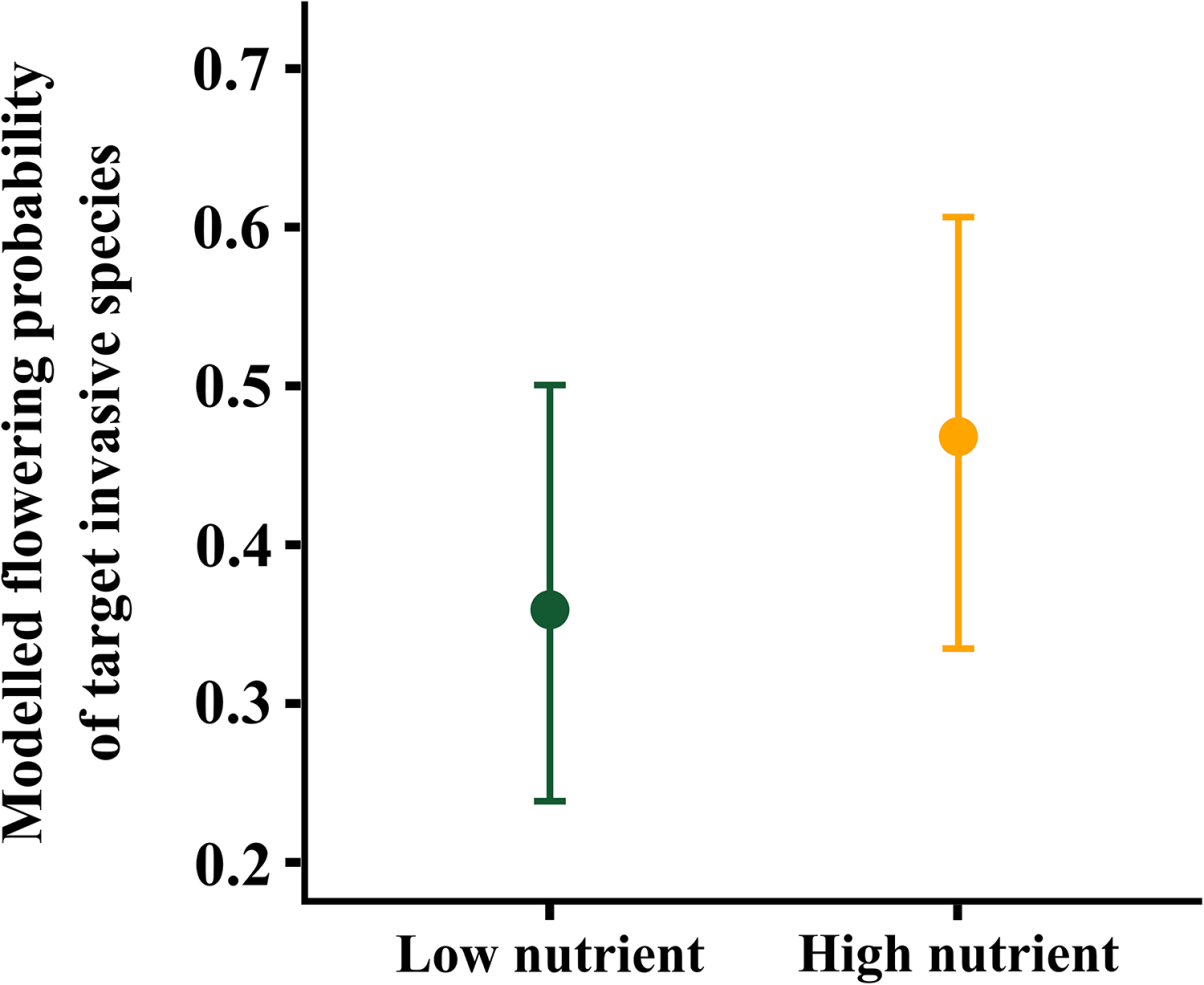
Modelled flowering probability of target invasive species under low- and high-nutrient enrichments. Points represent posterior means, and error bars represent 95% credible intervals.

## 4. Discussion

Support for our three predictions was limited and varied among response variables. For the first hypothesis, the interactive effect of ALAN and nutrient enrichment was supported only for proportional above-ground biomass: ALAN reduced the target invaders’ share of community biomass under low nutrients but increased it under high-nutrient conditions. The corresponding interactive effects on absolute above-ground biomass and flowering probability were uncertain, indicating that the proposed synergy was not consistently expressed across all dimensions of target invasive-plant performance. The effects of native species richness on each measured dimension of target invasive-plant performance were also unclear, providing no clear support for the second hypothesis. Likewise, the extent to which native species richness modified target invasive-plant responses to ALAN, nutrient enrichment or their combination remained uncertain, providing no clear support for the third hypothesis. The absence of a clear richness effect suggests that species number alone may not adequately represent biotic resistance, which may depend more strongly on community composition, functional traits, resident biomass and establishment history [44,62–65]. Initial height of target invasive species was positively associated with their above-ground biomass and proportional above-ground biomass and negatively associated with native-community biomass, whereas its association with flowering probability remained uncertain. These relationships are consistent with initially taller plants retaining an advantage through size-asymmetric competition for light by intercepting more light and shading smaller competitors [66,67]. Overall, invasion outcomes were primarily associated with nutrient-dependent shifts in relative performance and initial size asymmetries rather than with ALAN–nutrient synergy or native richness, with growth, flowering and dominance responding as distinct dimensions of invasion.

### (a) Nutrient enrichment increased both invasive and native biomass but did not clearly increase invasive-plant dominance

Nutrient enrichment exerted the strongest positive effect on the absolute above-ground biomass of target invasive species, increasing above-ground production by 272.88%. This result is consistent with evidence that invasive plants often respond positively to increased nutrient availability [5,14,68,69]. Elevated nutrient supply can relax below-ground resource limitation, allowing resource-acquisitive plants to attain high relative growth rates and allocate more biomass above-ground, thereby enhancing light capture and competitive effects [70]. However, nutrient enrichment also increased native-community above-ground biomass by 97.58%, indicating that both invasive and native plants benefited. Although the percentage increase was numerically greater for target invasive plants, the posterior mean effect on proportional invasive biomass was positive, with a 95% credible interval that slightly overlapped zero, providing no clear evidence that nutrient enrichment shifted the competitive balance towards invaders. Nutrient-driven gains in native biomass are common in resource-limited systems [71,72], and concurrent responses of native and invasive plants may increase overall community productivity without increasing proportional invader dominance [73]. Thus, the findings support the fluctuating resource hypothesis insofar as greater resource availability stimulated plant growth [9], but they do not demonstrate that eutrophication necessarily favoured invasive plants over native communities. Assessments based solely on absolute invasive-plant biomass may therefore overestimate the community-level effects of nutrient enrichment; invader performance should also be evaluated relative to that of resident plants.

### (b) ALAN and nutrient enrichment interacted to shape invasive plant dominance within native communities

ALAN altered the proportional above-ground biomass of target invasive species within native communities without consistently affecting their absolute biomass; it suppressed their share of community biomass under low-nutrient conditions but facilitated their dominance under high-nutrient conditions (figure 1b). This nutrient-dependent reversal reflected contrasting biomass responses of the target and resident components of the community. ALAN increased native-community biomass under both nutrient levels, but the increase was substantially greater under low-nutrient (29.75%) than under high-nutrient (13.10%) conditions (figure 2c). Under low nutrients, this strong resident response increased native biomass relative to target biomass, thereby reducing the invaders’ proportional contribution and potentially strengthening biotic resistance. Under high-nutrient conditions, the smaller increase in native-community biomass coincided with a greater proportional contribution of invaders, despite the absence of a consistent overall effect on their absolute biomass. Thus, ALAN changed the target invaders’ share of community biomass not by consistently increasing their own biomass, but by eliciting different responses from the target invasive and native components under low- and high-nutrient conditions. This context dependence is consistent with resource co-limitation, whereby nutrient availability influences which community members can translate additional nocturnal irradiance into growth and competitive advantage. Differences in light-use efficiency, nutrient-use efficiency, canopy position or biomass allocation could underlie these contrasting responses, although these traits were not measured. Previous studies similarly show that ALAN interacts with nutrient supply, water availability, plant density and herbivory to alter plant performance and competition [8,18,74,75]. Accordingly, ALAN may either strengthen resident resistance or create an invasion window, depending on the nutrient environment and the relative resource-acquisition responses of target and resident plants [9].

Although resource co-limitation provides a plausible explanation for the nutrient-dependent shift in invasive dominance observed here, the physiological processes and response traits underlying this interaction remain unresolved. ALAN can alter photosynthesis, respiration, carbohydrate metabolism, stomatal behaviour, circadian regulation and biomass allocation, but the magnitude and direction of these responses vary among species and environmental contexts [24,75]. We quantified treatment outcomes using above-ground biomass, flowering probability and proportional biomass, which represent vegetative performance, reproductive performance and community-level dominance, respectively, rather than the functional traits or physiological processes producing those outcomes. We also did not quantify actual resource limitation or physiological processes within the communities. Consequently, we cannot determine which response traits mediated treatment effects or whether the observed variation was structured more strongly by functional strategy than by invasion status. Nevertheless, species-specific posterior estimates of ALAN effects on proportional target biomass varied in both direction and magnitude, suggesting that functional differences among species may have contributed to the community-level pattern. Resolving these mechanisms will require measurements of night-time carbon assimilation, dark respiration, stomatal conductance, nutrient uptake, specific leaf area, leaf nitrogen concentration, canopy height, phenology and biomass allocation in both target and native plants. Such data would permit community-weighted trait analyses and direct tests of whether functional traits predict responses to ALAN and nutrient enrichment more effectively than invasive–native status.

The experimental illumination regime provides a clearly defined ecological context for interpreting these mechanisms. Our ALAN treatment simulated direct exposure to streetlight-like illumination at approximately 30.5 lux, providing direct insight into plant responses in highly illuminated microsites, such as roadsides, park edges, building margins and vegetation adjacent to artificial lights. Urban vegetation also experiences a broader range of weaker, diffuse and spatially heterogeneous illumination, including reflected light and skyglow; ecological responses may therefore vary along these lighting gradients. Building on our findings, field studies could cross realistic gradients of night-time illuminance and spectral composition with nutrient availability while measuring species-level traits and performance. Such studies would broaden the ecological scope of our results, establish the spatial extent of these responses and determine whether trait-mediated shifts in competitive dominance persist under field conditions.

### (c) Nutrient enrichment promoted reproductive initiation, whereas ALAN effects were inconsistent

Nutrient enrichment increased the flowering probability of target invasive plants by 30.39% (figure 3), suggesting that greater nutrient availability allowed more individuals to reach the developmental threshold for reproduction. Similar positive effects of nutrient enrichment on invasive-plant reproduction have been reported in both single-species and multispecies experiments [73,76]. Our binary measure, based on the presence of buds, flowers or fruits, therefore captured entry into the reproductive phase, an important life-history transition, rather than subsequent reproductive output. Accordingly, this result demonstrates enhanced reproductive initiation, while direct measures of reproductive output are needed to determine whether this response increases fecundity. Flowering probability, however, showed no consistent response to either the main effect of ALAN or the ALAN × nutrient interaction. Nutrient enrichment therefore emerged as the principal experimental driver of reproductive initiation, whereas biomass responses reflected both nutrient enrichment and nutrient-dependent ALAN effects. This divergence indicates that environmental drivers can differentially affect vegetative and reproductive performance: conditions that enhance biomass accumulation or alter competitive dominance need not accelerate the transition to reproduction over the same time scale. Although not measured here, interspecific variation in photoperiodic and spectral sensitivity may have generated heterogeneous ALAN responses among the eight target species that did not resolve into a consistent overall flowering response. Longer experiments should therefore quantify flowering onset and duration, flower and fruit production, seed quantity and viability, and recruitment in both target and resident species across gradients of ALAN intensity and spectral composition crossed with nutrient availability. Linking these reproductive measures to species-level light-response traits would clarify when ALAN-induced phenological shifts translate into altered propagule pressure and subsequent community assembly.

### (d) No clear effects of native species richness on invasive-plant performance or its responses to ALAN and nutrients

Contrary to our hypotheses, there was no clear evidence that native species richness affected target invasive-plant biomass, proportional invasive biomass or flowering probability. Nor did we find clear evidence that richness modified target invasive-plant responses to ALAN and nutrient enrichment. Thus, this simultaneous-assembly experiment provided no clear support for richness-based biotic resistance through niche occupation, resource pre-emption or competitive exclusion [38,45,62], or for its weakening by ALAN and nutrient enrichment. This outcome does not imply that diversity is unimportant in established communities because the target invasive species and native communities were established simultaneously, rather than the invaders being introduced into pre-established native communities. Several design features may have limited the expression of richness effects. Native density was held constant across richness treatments, and all plants were established simultaneously in homogeneous substrate; increasing richness therefore altered community composition and individual species’ abundances but may not have increased total resource capture or niche occupancy. The short experimental duration may also have restricted priority effects, resident-biomass accumulation, resource pre-emption and partitioning and plant–soil microbial legacies, all of which can strengthen resistance as communities mature [41,77,78]. The experiment consequently tested diversity effects during early community assembly rather than the resistance of established vegetation. Community composition and functional structure may also govern resistance more strongly than species number. Species-poor assemblages containing strong or functionally distinct competitors may resist invasion as effectively as richer assemblages dominated by weak or functionally redundant species. Accordingly, dominant-species identity, functional-group composition, resident–invader trait differences and functional or phylogenetic diversity can predict resistance more effectively than taxonomic richness alone [44,64,65,77]. Diversity–invasibility relationships also vary with spatial scale and environmental context, including reversals described by the invasion paradox [79]. Although our experiment did not directly test these mechanisms, the findings show that taxonomic richness alone did not clearly capture the community attributes mediating invasion or responses to ALAN and nutrient enrichment. Experiments that independently manipulate richness, composition, functional diversity, resident biomass and establishment history while factorially crossing ALAN and nutrient supply would clarify when diversity confers resistance under interacting global-change drivers.

## 5. Conclusion

Nutrient enrichment was the strongest driver of target invasive-plant performance, increasing absolute above-ground biomass and the probability of reproductive initiation, but it also increased native-community biomass. Greater nutrient availability therefore enhanced community productivity without clearly shifting the competitive balance towards target invaders. ALAN showed no consistent independent effect on target invasive-plant biomass or flowering probability but altered proportional invasive biomass in a nutrient-dependent manner, reducing the target invaders’ share of community biomass under low nutrients but increasing it under high-nutrient conditions. Consistent with the fluctuating resource hypothesis, this reversal suggests that the consequences of night-time illumination depend on the surrounding nutrient environment and that interacting global-change drivers cannot be inferred from their independent effects. We found no clear evidence that native species richness affected target invasive-plant performance or modified its responses to ALAN and nutrient enrichment during the simultaneous establishment of target invasive species and native communities. Within this experimental context, invasion outcomes were more closely associated with resource availability and relative biomass responses than with taxonomic richness, while the potential contributions of species identity, functional composition, resident biomass and establishment history remain to be tested. Overall, predicting and managing invasion in increasingly eutrophic and light-polluted environments requires evaluating interacting drivers across distinct dimensions of invasion, including absolute growth, reproductive initiation and dominance relative to native communities.

## Supplementary Material



## Data Availability

The data that support the findings of this study are openly available via Figshare at [80].
