# Supplementary material for: Artificial light at night alters the relative dominance of invasive plants in a nutrient-dependent manner across native species richness levels
Source: Proc Biol Sci. 2026 Oct 28;293(2081):20260507. doi: 10.1098/rspb.2026.0507 (PMC13725529; doi:10.1098/rspb.2026.0507)
Supplement: Supplementary file 1 [file rspb.2026.0507.zip › rspb.2026.0507/rspb20260507_si_001.pdf]

Electronic supplementary material for "Artificial light at night alters the relative dominance of invasive plants in a nutrient-dependent manner across native species richness levels"

Haobin Zhang[1], Ayub M. O. Oduor[1,2,3], Lu Xiao[1*], Yanjie Liu[1*]

[1]Jilin Da'an Agro-ecosystem National Observation and Research Station, Key Laboratory of Marsh Wetland Ecosystem Conservation and Restoration, National Forestry and Grassland Administration, State Key Laboratory of Black Soils Conservation and Utilization, Northeast Institute of Geography and Agroecology, Chinese Academy of Sciences, Changchun, Jilin, People's Republic of China

[2]Department of Biological and Life Sciences, Technical University of Kenya, Nairobi, Kenya

[3]Shandong Key Laboratory of Eco-Environmental Science for Yellow River Delta, Shandong University of Aeronautics, Binzhou, Shandong, People's Republic of China

*Correspondence: Lu Xiao, xiaolu@iga.ac.cn; Yanjie Liu, liuyanjie@iga.ac.cn

Table S1. Details of the eight invasive non-native and 10 native species used in the experiment.

Species	Family	Invasive status	Seed source	Sowing date
Ambrosia artemisiifolia L.	Asteraceae	Invasive	Inner Mongolia, China	2023.8.27
Ambrosia trifida L.	Asteraceae	Invasive	Jilin, China	2023.8.27
Amaranthus retroflexus L.	Amaranthaceae	Invasive	Jilin, China	2023.8.27
Bidens pilosa L.	Asteraceae	Invasive	Jilin, China	2023.8.30
Cenchrus longispinus (Hack.) Fernald	Poaceae	Invasive	Jilin, China	2023.8.27
Lolium perenne L.	Poaceae	Invasive	Green Alliance Ecological Enterprise Shop, Changde, Hunan, China	2023.8.27
Medicago sativa L.	Fabaceae	Invasive	Inner Mongolia, China	2023.8.30
Hibiscus trionum L.	Malvaceae	Invasive	Jilin, China	2023.8.27
Artemisia sieversiana Ehrh. ex Willd.	Asteraceae	Native	Inner Mongolia, China	2023.8.20
Artemisia scoparia Waldst. & Kit.	Asteraceae	Native	Inner Mongolia, China	2023.8.20
Poa annua L.	Poaceae	Native	Inner Mongolia, China	2023.8.20
Galium verum L.	Rubiaceae	Native	Jilin, China	2023.8.20
Sanguisorba officinalis L.	Rosaceae	Native	Jilin, China	2023.8.20
Potentilla tanacetifolia Willd. ex Schltdl.	Rosaceae	Native	Inner Mongolia, China	2023.8.20
Leonurus artemisia (Lour.) S. Y. Hu	Lamiaceae	Native	Inner Mongolia, China	2023.8.20
Lepidium apetalum Willd.	Brassicaceae	Native	Jilin, China	2023.8.20
Astragalus adsurgens Pall.	Fabaceae	Native	Inner Mongolia, China	2023.8.21
Suaeda salsa (L.) Pall.	Amaranthaceae	Native	Jilin, China	2023.8.21

Table S2. Candidate Bayesian hierarchical models, model comparisons, and diagnostic summaries.

Response variable	Model	Fixed-effect structure	Species-level random structure	Greenhouse compartment included	elpd_diff	se_diff	max Pareto k	max Rhat	min Bulk ESS	min Tail ESS	Divergences	Selected
Above-ground biomass of target invasive species	R1	C × L × N + H	(1 | Species)	Yes	-54.932	11.230	0.450	1.001	5209	7171	0	No
	R2	C × L × N + H	(1 + L || Species)	Yes	-54.802	11.215	0.404	1.001	4695	7510	0	No
	R3	C × L × N + H	(1 + N || Species)	Yes	0.000	0.000	0.491	1.001	5248	6999	0	Yes
	R4	C × L × N + H	(1 + L + N || Species)	Yes	-0.045	0.236	0.441	1.001	5650	8852	0	No
	R5	C × L × N + H	(1 + L + N + L × N || Species)	Yes	-0.535	0.988	0.623	1.001	4919	7455	0	No
	F1	C × L × N + H	Selected R3 structure	Yes	-3.286	1.505	0.491	1.001	5248	6999	0	No
	F2	C × L + C × N + L × N + H	Selected R3 structure	Yes	-2.310	1.229	0.463	1.001	5362	7964	0	No
	F3	C + L × N + H	Selected R3 structure	Yes	-0.279	1.109	0.476	1.001	5172	7509	0	No
	F4	C + L + N + H	Selected R3 structure	Yes	0.000	0.000	0.498	1.001	6003	9714	0	Yes
Proportional above-ground biomass of target invasive species	R1	C × L × N + H	(1 | Species)	Yes	-60.655	10.970	0.501	1.001	5234	7786	0	No
	R2	C × L × N + H	(1 + L || Species)	Yes	-60.782	10.956	0.436	1.001	5209	7244	0	No
	R3	C × L × N + H	(1 + N || Species)	Yes	0.000	0.000	0.574	1.001	5084	7243	0	Yes
	R4	C × L × N + H	(1 + L + N || Species)	Yes	-0.097	0.215	0.482	1.001	5464	8271	0	No
	R5	C × L × N + H	(1 + L + N + L × N || Species)	Yes	-0.214	1.705	0.482	1.001	5663	8119	0	No
	F1	C × L × N + H	Selected R3 structure	Yes	-2.741	1.034	0.574	1.001	5084	7243	0	No

Table S2 continued

Response variable	Model	Fixed-effect structure	Species-level random structure	Greenhouse compartment included	elpd_diff	se_diff	max Pareto k	max Rhat	min Bulk ESS	min Tail ESS	Divergences	Selected
Proportional above-ground biomass of target invasive species	F2	C × L + C × N + L × N + H	Selected R3 structure	Yes	-2.337	0.388	0.558	1.001	5015	7618	0	No
	F3	C + L × N + H	Selected R3 structure	Yes	0.000	0.000	0.489	1.001	4890	7684	0	Yes
	F4	C + L + N + H	Selected R3 structure	Yes	-2.977	3.058	0.556	1.001	7170	9700	0	No
Above-ground biomass of native communities	R1	C × L × N + H	(1 | Species)	Yes	-38.506	9.314	0.547	1.001	4637	5280	0	No
	R2	C × L × N + H	(1 + L || Species)	Yes	-38.537	9.325	0.432	1.002	3545	3851	0	No
	R3	C × L × N + H	(1 + N || Species)	Yes	-0.025	0.205	0.641	1.002	4991	6201	0	No
	R4	C × L × N + H	(1 + L + N || Species)	Yes	0.000	0.000	0.513	1.001	5199	6554	0	Yes
	R5	C × L × N + H	(1 + L + N + L × N || Species)	Yes	-0.727	0.613	0.552	1.001	4802	4204	0	No
	F1	C × L × N + H	Selected R4 structure	Yes	-2.644	1.157	0.610	1.001	3843	3617	0	No
	F2	C × L + C × N + L × N + H	Selected R4 structure	Yes	-1.609	0.914	0.567	1.002	3185	3454	0	No
	F3	C + L × N + H	Selected R4 structure	Yes	0.000	0.000	0.506	1.001	3795	4664	0	Yes
	F4	C + L + N + H	Selected R4 structure	Yes	-6.424	3.859	0.652	1.002	4588	5144	0	No

Table S2 continued

Response variable	Model	Fixed-effect structure	Species-level random structure	Greenhouse compartment included	elpd_diff	se_diff	max Pareto k	max Rhat	min Bulk ESS	min Tail ESS	Divergences	Selected
Flowering probability of target invasive species	R1	C × L × N + H	(1 | Species)	Yes	-2.388	2.950	0.623	1.001	2278	2061	0	No
	R2	C × L × N + H	(1 + L || Species)	Yes	-2.209	2.961	0.675	1.005	1780	1731	0	No
	R3	C × L × N + H	(1 + N || Species)	Yes	-0.451	0.691	0.688	1.002	2387	2762	0	No
	R4	C × L × N + H	(1 + L + N || Species)	Yes	-0.075	0.689	0.693	1.001	1933	1572	0	No
	R5	C × L × N + H	(1 + L + N + L × N || Species)	Yes	0.000	0.000	0.681	1.001	2407	1765	0	Yes
	F1	C × L × N + H	Selected R5 structure	Yes	-1.276	1.608	0.692	1.001	1391	746	0	No
	F2	C × L + C × N + L × N + H	Selected R5 structure	Yes	-1.057	1.074	0.686	1.001	1734	1199	0	No
	F3	C + L × N + H	Selected R5 structure	Yes	0.000	0.000	0.588	1.002	2313	1502	0	Yes
	F4	C + L + N + H	Selected R5 structure	Yes	-0.974	0.613	0.628	1.002	2791	1879	0	No
Total above-ground biomass per pot	R1	C × L × N + H	(1 | Species)	Yes	-22.479	6.822	0.469	1.002	4317	5086	0	No
	R2	C × L × N + H	(1 + L || Species)	Yes	-22.112	6.797	0.527	1.001	3512	4329	0	No
	R3	C × L × N + H	(1 + N || Species)	Yes	-0.287	0.280	0.508	1.001	4547	6387	0	No
	R4	C × L × N + H	(1 + L + N || Species)	Yes	0.000	0.000	0.559	1.001	4345	4910	0	Yes
	R5	C × L × N + H	(1 + L + N + L × N || Species)	Yes	-0.787	0.763	0.470	1.002	3568	4315	0	No

Table S2 continued

Response variable	Model	Fixed-effect structure	Species-level random structure	Greenhouse compartment included	elpd_diff	se_diff	max Pareto k	max Rhat	min Bulk ESS	min Tail ESS	Divergences	Selected
Total above-ground biomass per pot	F1	C × L × N + H	Selected R4 structure	Yes	-2.082	1.566	0.564	1.001	3583	4375	0	No
	F2	C × L + C × N + L × N + H	Selected R4 structure	Yes	-0.809	1.608	0.452	1.001	3952	4909	0	No
	F3	C + L × N + H	Selected R4 structure	Yes	0.000	0.000	0.567	1.001	3355	3641	0	Yes
	F4	C + L + N + H	Selected R4 structure	Yes	-1.557	2.307	0.436	1.002	4107	5117	0	No

Note. C, native-community species richness; L, ALAN treatment; N, nutrient enrichment; H, standardized initial height of the target invasive species. Native-community species richness was coded as a continuous predictor with three richness levels (2, 4 and 8 native species). R1-R5 denote candidate species-level random-effect structures compared under the full fixed-effect structure. F1-F4 denote candidate fixed-effect structures compared after selecting the species-level random-effect structure for each response variable. The notation || indicates that correlations between random intercepts and random slopes were not estimated. elpd_diff and se_diff were obtained from approximate leave-one-out cross-validation and are reported relative to the best-performing model within each response variable. Rhat, bulk ESS, tail ESS, Pareto-k and divergent transitions were used to assess model reliability. Final selected models are indicated in the "Selected" column. Bayesian hierarchical models were fitted using *brms* [57], and approximate leave-one-out cross-validation was used for model comparison [59]. All candidate models included native-community identity and greenhouse-compartment identity as random effects.

References cited in Table S2

57. Bürkner, P.-C. 2017 brms: An R Package for Bayesian Multilevel Models Using Stan. J. Stat. Softw. 80, 1-28. (doi:10.18637/jss.v080.i01)

59. Vehtari, A., Gelman, A. & Gabry, J. 2017 Practical Bayesian model evaluation using leave-one-out cross-validation and WAIC. Stat. Comput. 27, 1413-1432. (doi:10.1007/s11222-016-9696-4)

Table S3. Results of the final Bayesian hierarchical model testing the effects of ALAN (No-ALAN vs. ALAN), nutrient enrichment (low vs. high) and native species richness (2, 4 or 8 species) on total above-ground biomass per pot. Posterior means, posterior standard deviations (s.d.), and the lower (LCrI) and upper (UCrI) bounds of the 95% credible intervals (CrIs) are presented. Boldface estimates indicate statistically significant effects, defined here as those for which the 95% CrIs exclude zero.

Term	estimate	posterior s.d.	L95%CrI	U95%CrI
Intercept (grand mean)	**1.367**	0.105	1.156	1.576
Initial height of target invasive species	**0.073**	0.019	0.037	0.110
ALAN (L)	**0.091**	0.028	0.036	0.146
Nutrient treatment (N)	**0.463**	0.035	0.391	0.531
Specie richness of native community (C)	0.000	0.010	-0.020	0.020
L: N	**-0.021**	0.009	-0.039	-0.002

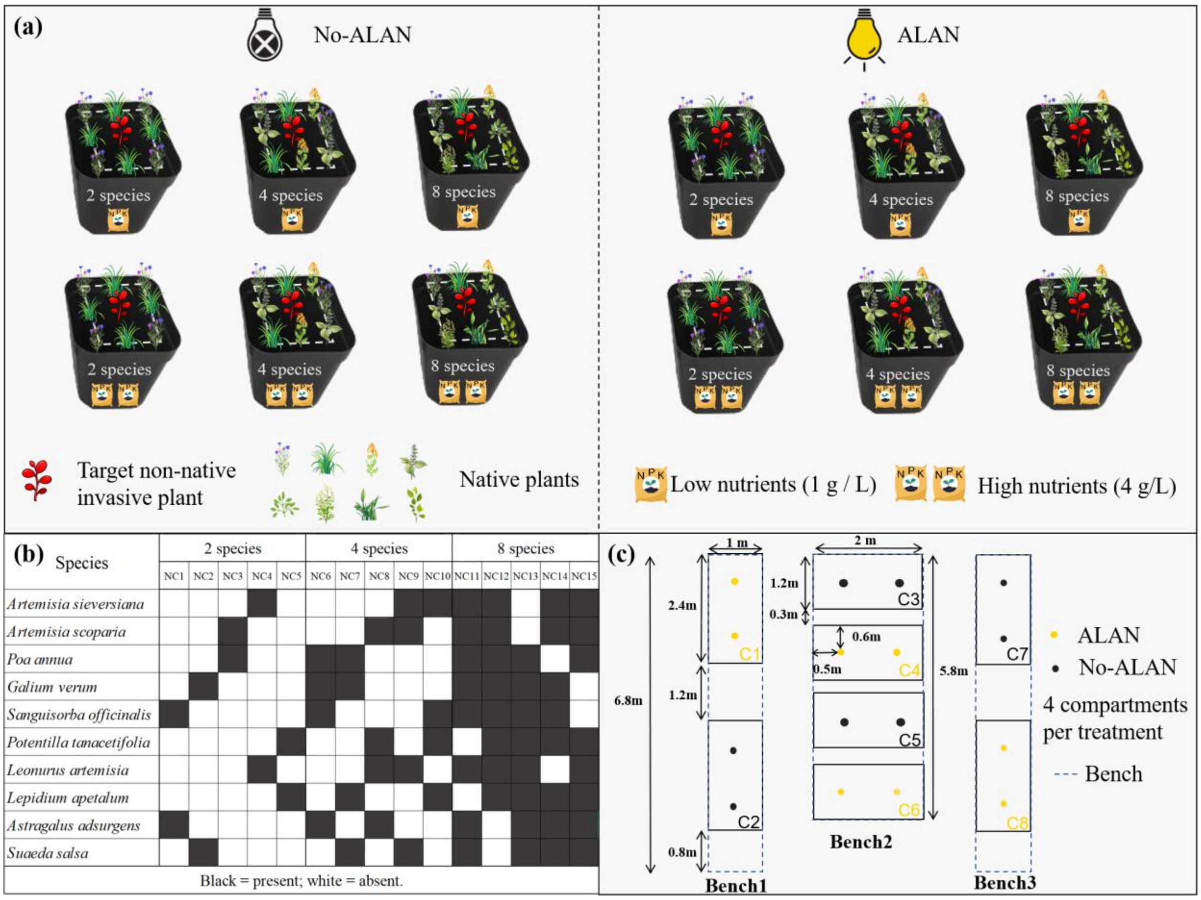

Figure S1. Schematic representation of the experimental design. (a) Fully factorial combinations of native species richness (2, 4 or 8 species), artificial light at night (No-ALAN or ALAN), and nutrient enrichment (low or high). Each pot contained one target invasive plant grown with a native community of eight individuals. (b) Species composition of the 15 native plant communities (NC1–NC15). Rows represent the 10 native species, and columns represent individual community compositions; black cells indicate species included in each community. Communities NC1–NC5, NC6–NC10 and NC11–NC15 correspond to the two-, four- and eight-species richness levels, respectively. (c) Spatial arrangement of the greenhouse compartments across the three benches. Yellow symbols denote spotlight positions in compartments assigned to the ALAN treatment; black symbols denote the corresponding positions in No-ALAN compartments. Compartments are labelled C1–C8. Red arrows in C4 indicate one spotlight at the centre of a 1 × 1.2 m sub-compartment.

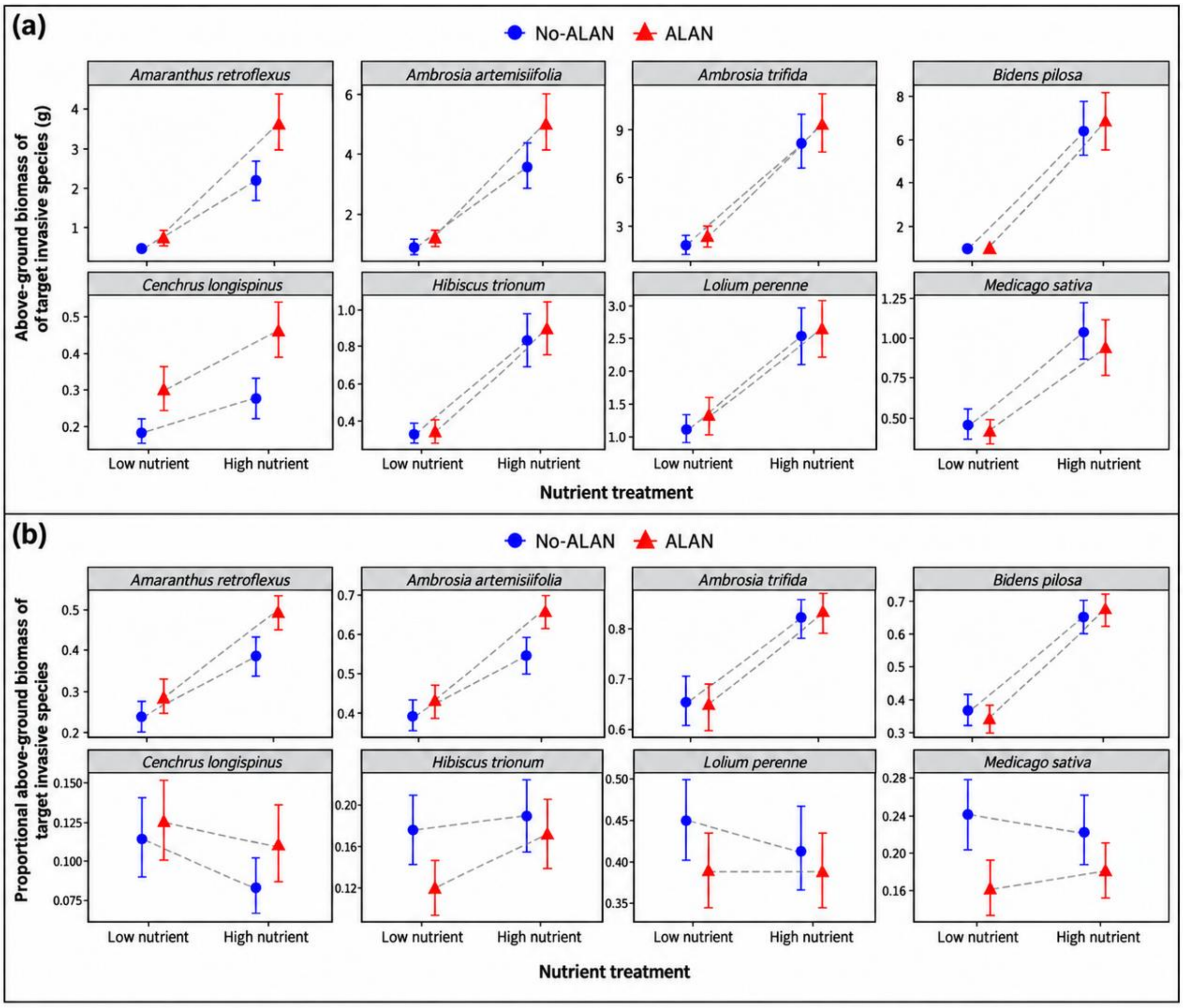

Figure S2. Species-specific modelled estimates of above-ground biomass and proportional above-ground biomass of the target invasive species under artificial light at night (No-ALAN or ALAN) and nutrient enrichment (low or high) treatments. Points represent posterior means, and error bars indicate 95% credible intervals.
